# THE ASSOCIATION BETWEEN NURSING HOME IT MATURITY AND UTIS AMONG LONG-TERM NH RESIDENTS

**DOI:** 10.1093/geroni/igac059.252

**Published:** 2022-12-20

**Authors:** Gregory Alexander

**Affiliations:** Columbia University, New York, New York, United States

## Abstract

Prof. Alexander will present a study linking national nursing home (NH) health information technology (HIT) to quality. This research explores relationships between NH HIT maturity and UTIs examined during a repeated cross-sectional study over 4 consecutive years (2013-2017). HIT maturity represents the diversity of technology use in NHs. Researchers conducted bivariate and multivariate regressions using MDS 3.0 resident assessments and post stratified survey weights controlling for NH characteristics. Our sample included 816 NHs. These NHs had 219,730 regular NH resident assessments within 90 days of a survey, representing 80,322 unique NH residents. Of these assessments, 4.1% recorded a UTI. In the multivariate analyses, administrative HIT capabilities was associated with lower odds of UTI (AOR: 0.906, 95% CI: 0.843, 0.973), controlling for covariates. Integration of administrative HIT systems may relieve the burden of tracing UTIs, data documentation and record retrieval affording NH staff time to focus their efforts on clinical care.

